# Inhibition of Wnt/β-Catenin Signaling in Neuroendocrine Tumors In Vitro: Antitumoral Effects

**DOI:** 10.3390/cancers12020345

**Published:** 2020-02-04

**Authors:** Xi-Feng Jin, Gerald Spöttl, Julian Maurer, Svenja Nölting, Christoph Josef Auernhammer

**Affiliations:** 1 Department of Internal Medicine 4, University-Hospital, Klinikum der Universitaet Muenchen, Ludwig-Maximilians-University of Munich, 81377 Munich, Germany; xifeng.jin@campus.lmu.de (X.-F.J.); gerald.spoettl@med.uni-muenchen.de (G.S.); Julian.Maurer@med.uni-muenchen.de (J.M.); Svenja.Noelting@med.uni-muenchen.de (S.N.); 2 Interdisciplinary Center of Neuroendocrine Tumors of the GastroEnteroPancreatic System (GEPNET-KUM), Klinikum der Universitaet Muenchen, Ludwig-Maximilians-University of Munich, Campus Grosshadern, Marchioninistr. 15, 81377 Munich, Germany

**Keywords:** neuroendocrine tumor, Wnt/β-catenin signaling, porcupine inhibitor, β-catenin inhibitor, β-catenin siRNA

## Abstract

Background and aims: Inhibition of Wnt/β-catenin signaling by specific inhibitors is currently being investigated as an antitumoral strategy for various cancers. The role of Wnt/β-catenin signaling in neuroendocrine tumors still needs to be further investigated. Methods: This study investigated the antitumor activity of the porcupine (PORCN) inhibitor WNT974 and the β-catenin inhibitor PRI-724 in human neuroendocrine tumor (NET) cell lines BON1, QGP-1, and NCI-H727 in vitro. NET cells were treated with WNT974, PRI-724, or small interfering ribonucleic acids against β-catenin, and subsequent analyses included cell viability assays, flow cytometric cell cycle analysis, caspase3/7 assays and Western blot analysis. Results: Treatment of NET cells with WNT974 significantly reduced NET cell viability in a dose- and time-dependent manner by inducing NET cell cycle arrest at the G1 and G2/M phases without inducing apoptosis. WNT974 primarily blocked Wnt/β-catenin signaling by the dose- and time-dependent downregulation of low-density lipoprotein receptor-related protein 6 (LRP6) phosphorylation and non-phosphorylated β-catenin and total β-catenin, as well as the genes targeting the latter (c-Myc and cyclinD1). Furthermore, the WNT974-induced reduction of NET cell viability occurred through the inhibition of GSK-3-dependent or independent signaling (including pAKT/mTOR, pEGFR and pIGFR signaling). Similarly, treatment of NET cells with the β-catenin inhibitor PRI-724 caused significant growth inhibition, while the knockdown of β-catenin expression by siRNA reduced NET tumor cell viability of BON1 cells but not of NCI-H727 cells. Conclusions: The PORCN inhibitor WNT974 possesses antitumor properties in NET cell lines by inhibiting Wnt and related signaling. In addition, the β-catenin inhibitor PRI-724 possesses antitumor properties in NET cell lines. Future studies are needed to determine the role of Wnt/β-catenin signaling in NET as a potential therapeutic target.

## 1. Introduction

Neuroendocrine tumors (NET) frequently occur in the gastroenteropancreatic system and lung [1,2]. The treatment strategies in inoperable advanced NETs depend on primary tumor location, grading, metastatic spread, hormonal secretion and genetics [3,4,5]. Established systemic treatment strategies include biotherapy with somatostatin analogs, chemotherapy with streptozotocin/5-fluorouracil or capecitabine/temozolomide in pancreatic NETs, peptide receptor-based radionuclide therapy (PRRT) with 177Lutetium-DOTA-TATE, and molecular targeted therapy with everolimus or sunitinib [3,4,5]. Despite our increasing understanding of the genetics and molecular biology of NETs [6,7], and growing preclinical and clinical data of novel molecular targeted therapy strategies and precision oncology approaches in NETs [8,9], there is still an unmet medical need for further novel systemic treatment strategies in advanced NETs [9,10]. In the current in vitro study, we aimed to investigate the potential role of Wnt/β-catenin signaling as a novel strategy of molecular targeted therapy in neuroendocrine tumor cells.

The Wnt/β-catenin signaling cascade is essential in various cancers [11,12,13]. The potential role of Wnt antibodies (e.g., OMP-18R5 (Vantictumab), OMP-54 F28 (Ipafricept), Foxy-5, OTSA 101), porcupine inhibitors (e.g., WNT974 (synonym LGK974), IWP-2, ETC-1922159), direct β-catenin inhibitors (e.g., PRI-724, CWP232291, PKF115-584, ICG-001, PKF118-310, NCB-0846), tankyrase inhibitors (e.g., IWR1, XAV939, NVP-TNKS656, JW74), and disheveled inhibitors (e.g., NSC668036, J01-017a) to target the Wnt/β-catenin signaling cascade in cancer [11,12,13] is currently being investigated. Several agents, including the porcupine inhibitor WNT974 and the β-catenin inhibitor PRI-724, have already entered clinical phase I/II studies [11,13]. Targeting Wnt/β-catenin signaling might also be important in the immunotherapy of cancer as Wnt/β-catenin signaling affects dendritic, CD8^+^ T, CD4^+^ T and regulatory T cells [14,15].

The potential role of Wnt/β-catenin signaling in neuroendocrine tumors (NETs) has recently been reviewed [9,16]. Wnt/β-catenin signaling has been demonstrated to play a role in NET tumor cell growth regulation in vitro and in vivo [16,17,18,19] and NET tumor cell invasion [20]. The multiple endocrine neoplasia MEN1 gene derived protein Menin seems to be a negative regulator of β-catenin [18]. In MEN1-deficient knockout mice, β-catenin is activated in pancreatic neuroendocrine tumors (pNETs) while conditional β-catenin knockout decreased tumorigenesis of pNETs in this in vivo model [18]. Analysis of human NET tumor samples demonstrated mutations of the MEN1 gene in up to 35% of lung carcinoids [21,22,23] and up to 37% of pNETs [6]. Mutations in other well-known negative regulators of the Wnt/β-catenin signaling cascade, such as the APC gene, were present in 6%–12% of typical/atypical lung carcinoids [24] and in 8–23% of SI-NETs [25,26]. Immunohistochemical studies of the Wnt/β-catenin signaling in human pNETs [27], lung carcinoids [23,28] and SI-NETs [19] have been reported.

The Wnt signaling pathway regulates gene expression, cell proliferation and migration during embryogenic development and tumorigenesis [29,30]. Wnt protein ligands bind to a Frizzled family receptor; to date, there are a total of 19 Wnt ligands and 10 Frizzled (FZD) receptors. The Wnt signaling pathways can be divided into: (i) canonical Wnt signaling, mediated by Frizzled (FZD)/LRP5/6 receptor and downstream β-catenin; (ii) non-canonical Wnt signaling (primarily initiated by Wnt5a/b and Wnt11), mediated by Frizzled (FZD) and co-receptors mediating downstream Wnt/planar cell polarity, Wnt/receptor tyrosine kinase, and Wnt/calcium signaling pathways. Specifically, the canonical Wnt signaling cascade is activated through the binding of secreted Wnt proteins (such as Wnt1 or Wnt3) to their membrane receptors, such as the FZD family of proteins and the low-density lipoprotein receptor-related protein (LRP) 5 or 6, which, in turn, leads to phosphorylation of the adaptor protein Dishevelled (DVL) and induction of the interaction of DVL with Axin, an inhibitor of the Wnt signaling pathway, to inhibit the enzymatic activity of glycogen synthase kinase 3β(GSK3β). Thereafter, the activated Wnt proteins will induce the expression of unphosphorylated β-catenin and translocation of β-catenin into the nucleus, where the activated β-catenin will bind to transcription factor/lymphoid enhancer-binding factor (TCF/LEF) to enhance gene transcription and expression, such as for cyclinD1 and LEF1 [12,29,30]. On the other hand, the non-canonical Wnt signaling cascade, including Wnt/receptor tyrosine kinase (RTK) signaling, activates the PI3K/-Akt signaling cascade through RTKs such as ROR1 and ROR2, in addition to further downstream mediators [12,29,30].

Porcupine (PORCN) is an O-acetyltransferase necessary for the palmitoylation and processing of Wnt ligands [31,32]. PORCN is an endoplasmic reticulum transmembrane protein [31,32]. To inhibit Wnt signaling, previous studies demonstrated the effective use of selective porcupine (PORCN) inhibitors, such as WNT974 [31,32]. The porcupine inhibitor WNT974 has been reported in both in vitro and in vivo preclinical cancer models to inhibit tumor cell growth as well as tumor invasion/metastasis [32,33,34,35,36], and to act as a chemosensitizer [37] or radiosensitizer [38]. By contrast, the β-catenin inhibitor PRI-724 directly targets the protein–protein interaction of β-catenin with the CREB-binding protein (CBP) to inhibit Wnt signaling in various cancer models [11,12,13,39,40,41,42,43]. The porcupine inhibitor WNT974 and the β-catenin inhibitor PRI-724 have already entered clinical phase I/II studies [11,13].

In the current study, we aimed to investigate the potential role of in vitro inhibition of Wnt/β-catenin signaling as a novel strategy of molecular targeted therapy in neuroendocrine tumor cells. We investigated the in vitro effects of the PORCN inhibitor WNT974 and the β-catenin inhibitor PRI-724 in human NET cells. We provide insightful information regarding the canonical and non-canonical Wnt signaling pathway in NET cells and demonstrate inhibition of Wnt/β-catenin signaling as a potential treatment strategy in NET.

## 2. Materials and Methods

### 2.1. Cell Lines, Culture, and Treatment

Human pancreatic NET cell line BON1 [9] (kindly provided by R. Göke, University of Marburg, Marburg, Germany) and pancreatic islet tumor cell line QGP-1 [9] (originally obtained from the Japanese Collection of Research Bioresources Cell Bank, Osaka, Japan), were both maintained in Dulbecco’s modified Eagle’s medium/F12 (at a ratio of 1:1) supplemented with 10% fetal bovine serum (FBS), 1% penicillin/streptomycin and 0.4% amphotericin B in a 37 °C humidified incubator with 5% CO_2_. Human bronchopulmonary neuroendocrine NCI-H727 tumor cells [9] (originally obtained from ATCC, Manassas, VA, USA) and the human small intestinal NET cell line GOT1 [9,39] (kindly provided by O. Nilsson, Sahlgrenska University Hospital, Göteborg, Sweden) were both cultured in Roswell Park Memorial Institute medium-1640 (RPMI-1640) supplemented with 10% FBS, 1% penicillin/streptomycin, and 0.4% amphotericin B in a 37 °C humidified incubator with 5% CO_2_.

To treat NET cells with WNT974 (also named LGK974; Novartis, Basel, Switzerland) or PRI-724 (Selleckchem, Germany), the cell lines were first seeded into cell culture dishes and grown overnight prior to treatment with various doses of WNT974 (1–32 µM, dissolved in dimethyl sulfoxide (DMSO)) or PRI-724 (1–10 µM, dissolved in DMSO) for different periods of time according to the assays listed below.

### 2.2. Cell Viability Assay and Population Doubling Time

To determine the effect of WNT974 or PRI-724 on the regulation of NET cell viability, we performed the Cell Titer Blue® cell viability assay (Promega, Madison, WI, USA). In particular, NET cells were grown overnight and then treated with various doses of WNT974 (1–32 µM) or PRI-724 (1–10 µM) for up to 144 h. At the end of each experiment, 20 µL of Cell Titer Blue^®^ solution was added to the cell culture followed by further culturing for 4 h. Thereafter, the fluorescence was measured at 560/590 nm using a GLOMAX plate reader (Promega, Madison, WI, USA).

The population doubling time (PDT) in the exponential growth phase was calculated using the formula: PDT = Δt × [ln2/(lnNt − lnN0)] [44]. All experiments, consisting of technical triplicates, were repeated at least three times. The data were expressed as a percentage of control as mean ± SD.

### 2.3. Flow Cytometric Cell Cycle Distribution Assay

NET cells were grown and treated with WNT974 (1–16 µM) for 72 h and then harvested through trypsinization, centrifugation and two washing steps in phosphate-buffered saline (PBS). After that, the cells were re-suspended in Nicoletti solution containing propidium iodide (Sigma-Aldrich, Taufkirchen, Germany) and then analyzed with the BD Accuri C6 flow cytometer and quantified using BD Accuri C6 Analysis software (BD Biosciences, Heidelberg, Germany) for cell cycle distribution. All experiments, consisting of technical triplicates, were repeated at least three times.

### 2.4. Caspase-3/7 Activity Assay

To assess changes in tumor cell apoptosis, we utilized the Apo-ONE Homogeneous Caspase-3/7 Assay (Promega, Mannheim, Germany). In brief, NET cells were seeded into 96 well plates at 10,000 cells per well and grown overnight, treated with 1 µM and 16 µM of WNT974 for 72 h, and then subjected to the Apo-ONE Homogeneous Caspase-3/7 Assay (Promega, Mannheim, Germany) according to the manufacturer’s instructions. The experiments, consisting of technical duplicates, were repeated at least three times.

### 2.5. Wound Healing Assay

NET cells were seeded into six-well plates containing cell culture inserts (Ibidi, Munich, Germany) at a density of 120,000–140,000 cells per chamber and grown for 24 h. Afterwards the cell culture inserts were removed, fresh medium with 1% FBS was added, and the cells were treated with WNT974 for 24 h. The wound gap (created with the cell culture inserts) was observed and photographed using a Zeiss Axiovert 135 TV microscope fitted with a Zeiss AxioCam MRm camera (Zeiss, München, Germany). The NET cell migration activity was calculated by measuring the relative gap at each time point with Image–J software (NIH, Bethesda, MD, USA). The experiments, consisting of technical duplicates, were repeated at least three times.

### 2.6. Protein Extraction and Western Blotting

NET cells were grown and then lysed in lysis buffer M-PER (Mammalian Protein Extraction Reagent) containing the HALT protease and phosphatase inhibitor cocktail from Thermo Scientific (Rockford, IL, USA). Cell protein was quantified, and for all Western blot experiments, samples were adjusted to the same protein concentration (30–50 µg/50 µL; Rotiquant Universal, Carl Roth, Karlsruhe, Germany). For Western blotting, the protein samples were first separated using sodium dodecyl sulfate–polyacrylamide gel electrophoresis (SDS-PAGE) gels and transferred onto polyvinylidene fluoride membranes (EMD Millipore, Billerica, MA, USA). Equal protein loading was verified in all Western blots by total protein staining and normalization, and using the housekeeping protein β-actin. After blocking in Clear Milk Blocking Buffer (PIERCE, Rockford, IL, USA) for 30 min, the membranes were incubated at 4 °C overnight with different primary antibodies. The used antibodies were against Axin-1, Dvl-2, pLRP6S1490, LRP6, SFRP, Wnt5a/b, cyclinD1, cyclinD3, cyclinB1, cdk1, cdk4, cdk6, Chk1, c-Myc, PCNA, vimentin, ZO-1 (zona occludens proteins), pEGFR Y1068, EGFR, non-pho-β-catenin S45, pho-β-catenin Ser33/37/Thr41, pho-β-catenin S552, total β-catenin, pIGFR Y1135, IGFR, pAkt S473, Akt, pmTOR S2448, pp70S6K T389, p70S6K, pERK1/2 T202/Y204, ERK1/2, pJNK T138/Y185, JNK, pGSK3 S21/9, and GSK3 (Cell Signaling Technology, Danvers, MA, USA); Bcl-2, and p21 Waf1/Cip1, (BD, Heidelberg, Germany); Menin and Neurotensin (Santa Cruz, Heidelberg, Germany); Wnt3a (abcam, Cambridge, UK); and β-actin, and p53 (Merck, Darmstadt, Germany). The following day, membranes were incubated with a horseradish peroxidase-conjugated secondary antibody (Cell Signaling Technology, Danvers, MA, USA) at a dilution of 1:25,000 for 2 h. The protein bands were visualized using a chemiluminescence Western blotting detection system (WESTAR Supernova, Cyanagen, Bologna, Italy). The chemiluminescence was detected by an imaging system (ECL Chemocam, INTAS, Göttingen, Germany) and quantification was performed using the gel macros of ImageJ (NIH, Bethesda, MD, USA).

### 2.7. siRNA and Cell Transfection

We obtained small interfering RNA (siRNA) pools containing four different siRNAs, each. The siRNA pools were as follows: β-catenin siRNA (ON-TARGETplus SMARTpool, cat #L-003482; Dharmacon, Lafayette, CO, USA), GSK3β siRNA (ON-TARGETplus SMARTpool, cat #L-003484; Dharmacon, Lafayette), or non-targeting control siRNA (ON-TARGET plus siCONTROL non-targeting pool, cat #D-001810; Dharmacon, Lafayette). To knockdown the expression of these genes, we grew NET cells overnight and then transfected them with 50 nM of each siRNA using DharmaFECT 2 or 3 transfection reagent (cat #T 2002-01 and cat #T 2003-01; Dharmacon, Lafayette) for 72 h. Thereafter, the cells were subjected to Western blot and other assays.

### 2.8. Statistical Analysis

Our data were summarized as the mean ± SD and statistically analyzed by one-way analysis of variance (ANOVA) or two-sample *t*-tests using SPSS 16.0 software for Windows (SPSS Inc., Chicago, IL, USA). A *p* value < 0.05 indicated statistical significance.

## 3. Results

### 3.1. WNT974 Reduces NET Cell Viability in a Dose- and Time-Dependent Manner

In pre-experiments, the population doubling time (PDT) was calculated as 0.895 ± 0.066 d for BON1, 1.536 ± 0.051 d for QGP-1, 1.781 ± 0.295 d for NCI-H727 cells and 15.48± 1.757 d for GOT1 cells respectively. Our results were in accordance with the short PDTs in BON1 and QGP-1 cells previously reported by Hofving et al [45], while the PDT of our GOT1 cells was even longer, with 15 days versus 5 days in the same report [45].

**Figure 1:**
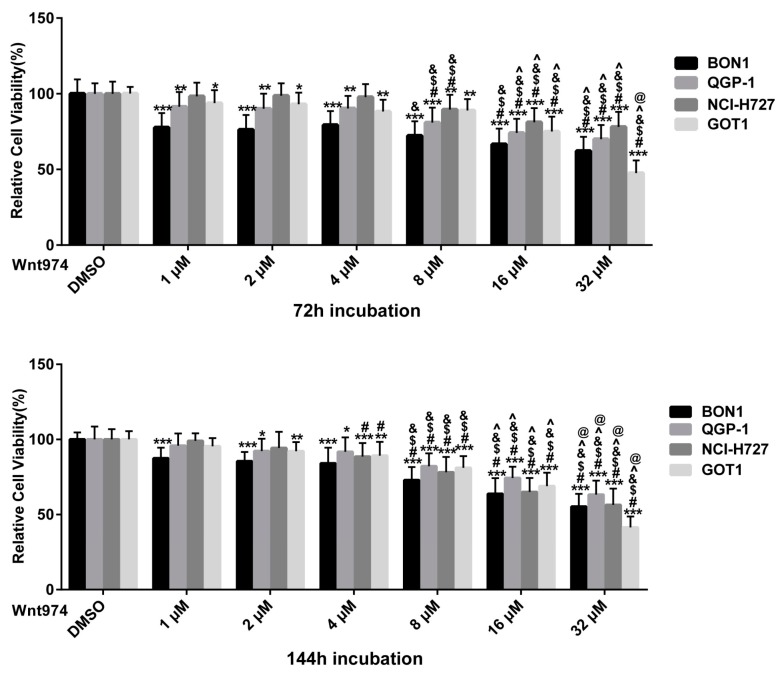
Effect of WNT974 on the reduction of neuroendocrine tumor (NET) cell viability in a dose- and time-dependent manner. The cell viability of human pancreatic BON1 and QGP-1, bronchial NCI-H727, and midgut GOT1 NET cell lines was assessed after treatment with WNT974 compared with that of dimethyl sulfoxide (DMSO) control. The data are expressed as mean ± SD. Each experiment, with technical triplicates, was repeated at least thrice. * *p* < 0.05, ** *p* < 0.01, and *** *p* < 0.001 compared with that of DMSO controls. ^#^ *p* < 0.05 (2 μM–32 μM vs. 1 μM, respectively), ^$^ *p* < 0.05 (4 μM–32 μM vs. 2 μM respectively), ^&^ *p* < 0.05 (8 μM–32 μM vs. 4 μM respectively), ^ *p* < 0.05 (16 μM–32 μM vs. 8 μM respectively), ^@^ *p* < 0.05 (16 μM vs. 32 μM).

Following the pre-experiments, we first assessed the effect of WNT974 (1–32 μM) on the regulation of cell viability. As shown in Figure 1, in the four cell lines BON1, QGP-1, NCI-H727, and GOT1, WNT974 treatment caused a dose- and time-dependent reduction of cell viability, i.e., after 144 h incubation at a dosage of 16 µM WNT974 with values of 63.8% ± 8.5% in BON1, 74.4% ± 7.4% in QGP-1, 65.0% ± 9.4% in NCI-H727, and 69.0% ± 8.9% in GOT1 cells. The calculated IC20 (concentration of drug which causes 20% inhibition of cell viability) value was 5.4 μM for BON1, 7.3 μM for GOT1, 7.8 μM for NCI-H727, and 10.1 μM for QGP-1.

Based on these observations, BON1 exhibited the most pronounced response to WNT974. Due to the long PDT of GOT1 cells, and, thus, their limited availability, all further experiments were performed using only BON1, QGP-1, and NCI-H727 cells.

### 3.2. WNT974 induces NET Cell Cycle Arrest at the G0/G1 Phase and G2 Phase, but does not Cause Apoptosis

**Figure 2:**
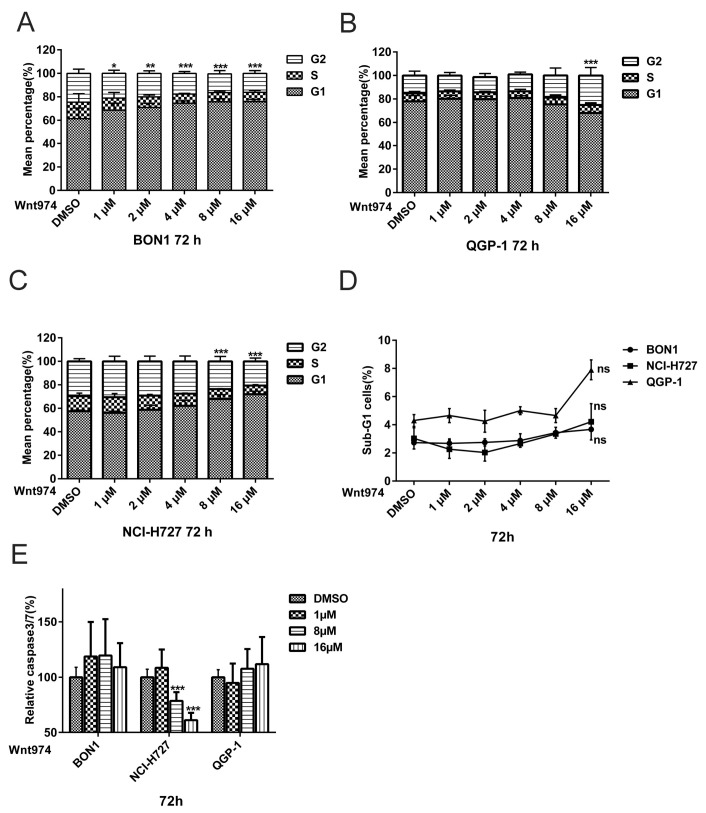
(**A**–**D**). Effect of WNT974 on cell cycle arrest at the G1, G2/M, and sub-G1 phase. BON1, QGP-1, and NCI-H727 cells were treated with or without WNT974 for 72 h and then subjected to flow cycle metric cell cycle distribution assay. The percentage of cells at each phase of the cell cycle is shown as the mean ± SD of three independent experiments. (**E**). Effect of WNT974 on the regulation of caspase-3/7 activity in NET cells. BON1, QGP-1, and NCI-H727 cells were treated with or without WNT974 for 72  h and then subjected to caspase-3/7 analysis, which shows the mean percentage of caspase-3/7 activity compared to the untreated control (100%) ± SD. * *p* < 0.05, ** *p* < 0.01, and *** *p* < 0.001 compared with that of DMSO control.

**Figure 3:**
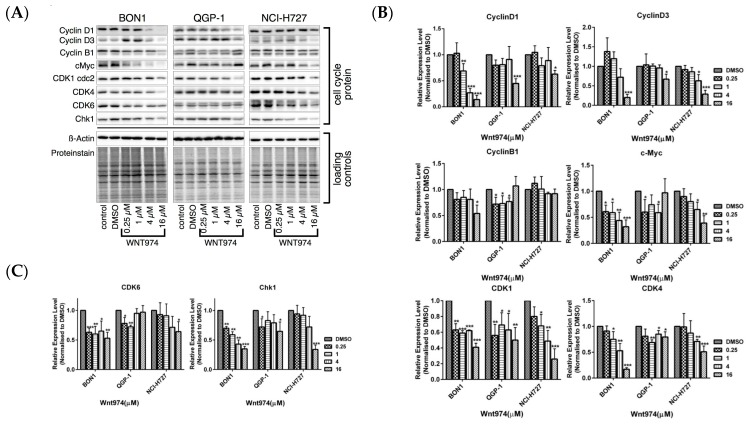
Effects of WNT974 on cell cycle proteins. BON1, QGP-1, and NCI-H727 cells were treated with or without WNT974 for 72 h and then subjected to Western blot analysis. (**A**) A representative Western blot is shown. Equal protein loading was verified in all Western blots by normalization to the total protein staining and by the housekeeping protein β-actin. (**B**,**C**) Densitometric quantification of Western blot data was performed. The DMSO control was set as 1.0. Relative expression levels (normalized to DMSO control) of treated cells were calculated in %. * *p* < 0.05, ** *p* < 0.01, and *** *p* < 0.001 compared with that of DMSO controls.

We next used FACS and Western blot to assess the effect of WNT974 treatment on the regulation of cell cycle distribution and apoptosis in order to better understand the WNT974-induced reduction of NET cell viability (Figure 2 and Figure 3). Treatment of NET cells with WNT974 at concentrations of 1–16 µM for 72 h resulted in the dose-dependent arrest of BON1 and NCI-H727 cells at the G1 phase of the cell cycle (Figure 2A,C). Following incubation with WNT974 (16 µM), 75.4% (vs. 61.4% of the control) and 74.0% (vs. 57.6% of the control) of the cells were observed to be in G1 phase for the BON1 and NCI-H727 cell lines, respectively. Meanwhile, the percentage of S phase cells decreased to 8.0% (vs. 14.1% of the control) and 7.4% (vs. 12.8% of the control), in BON1 and NCI-H727 cell lines, respectively. In QGP-1 cells, incubation with WNT974 (16 µM) induced the accumulation of cells in the G2 phase 25.13% (vs. 15.24% of the control cells) and subsequently decreased the percentage of cells in the G0/G1 phase to 68.07% (vs. 77.93% of the control cells) (Figure 2B). Thus, WNT974 caused cell cycle arrest in BON1, QGP-1, and NCI-H727 cells (Figure 2A–C). Accordingly, Western blot data demonstrated that treatment with WNT974 caused dose-dependent downregulation of the expression of cyclin D1, cyclin D3, and cyclin B1, as well as cyclin-dependent kinases (CDK1, CDK4, and CDK6) and checkpoint kinase-1 (CHK1) (Figure 3).

No induction of apoptosis was observed following incubation of BON1, QGP-1, and NCI-H727 cells with WNT974 at concentrations of 1–16 µM (Figure 2). Following WNT974 treatment, the FACS analysis showed no significant increase in sub-G1 phase accumulation (Figure 2D) and the caspase 3/7 assay showed no significant increase in caspase 3/7 activities (Figure 2E). Surprisingly, WNT974 even suppressed caspase 3/7 activity in NCI-H727 (*p* < 0.001).

In summary, WNT974 induced NET cell cycle arrest at the G1/G2 phase of the cell cycle but did not induce apoptosis.

### 3.3. Effects of WNT974 on the Inhibition of Wnt/β-Catenin Signaling in NET Cells

**Figure 4:**
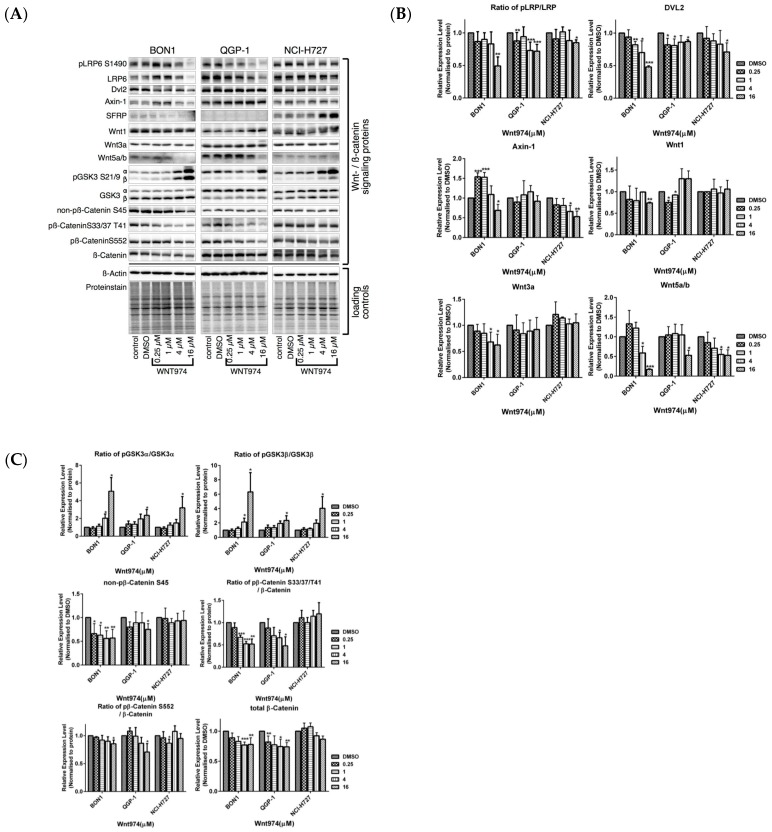
Effects of WNT974 on inhibition of the Wnt/β-catenin signaling in NET cells. BON1, QGP-1, and NCI-H727 cells were treated with or without WNT974 for 72  h and then subjected to Western blot analysis. (**A**) A representative Western blot is shown. Equal protein loading was verified in all Western blots by normalization to the total protein staining and by the housekeeping protein β-actin. (**B**,**C**) Densitometric quantification of Western blot data was performed. The DMSO control was set as 1.0. Relative expression levels (normalized to DMSO control) of treated cells were calculated in %. * *p* < 0.05, ** *p* < 0.01, and *** *p* < 0.001 compared with that of DMSO controls.

Treatment of BON1 and QGP-1 cells with WNT974 at concentrations of 0.25–16 µM significantly downregulated the expression of LRP/pLRP6, DVL2, and Wnt5a/b and reduced the level of non-phosphorylated β-catenin, total β-catenin, and β-catenin phosphorylation at Ser33/Ser37/Thr41 (Figure 4) and its downstream targeting proteins, such as c-Myc, cyclinD1, and cyclinD3 (Figure 3). These effects of WNT974 were more prominent in BON1 and QGP-1 cells but slightly weaker effects were also observed for NCI-H727 cells. However, secreted frizzled-related protein 1 (SFRP1), an antagonist of Wnt signaling, was increased by WNT974 treatment only in NCI-H727 cells (Figure 4), suggesting cell-line-specific modulation of the Wnt signaling cascade.

### 3.4. Effects of WNT974 on the Inhibition of the pAKT/mTOR, MAPK/ERK, pEGFR and pIGFR Pathways in NET Cells

Because the inhibitory effects of WNT974 on canonical Wnt/β-catenin signaling in NET cells were not unique in all three NET cell lines, we further assessed whether inhibition of non-canonical Wnt/receptor tyrosine kinase signaling, i.e., PI3K/-Akt/-mTOR, could mediate the effect of WNT974.

**Figure 5:**
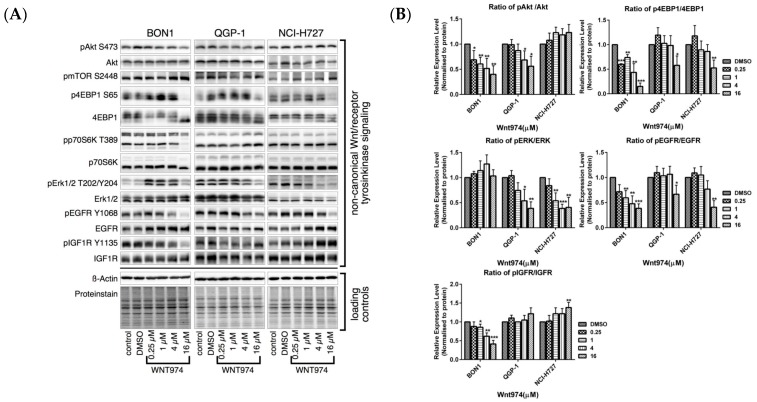
Effects of WNT974 on the inhibition of the pAKT/mTOR, MAPK/ERK, pEGFR and pIGFR pathways in NET cells. BON1, QGP-1, and NCI-H727 cells were treated with or without WNT974 for 72 h and then subjected to Western blot analysis. (**A**) A representative Western blot is shown. Equal protein loading was verified in all Western blots by normalization to the total protein staining and by the housekeeping protein β-actin. (**B**) Densitometric quantification of Western blot data was performed. The DMSO control was set as 1.0. Relative expression levels (normalized to DMSO control) of treated cells were calculated in %. * *p* < 0.05, ** *p* < 0.01, and *** *p* < 0.001 compared with that of DMSO controls.

Treatment of NET cells with WNT974 at concentrations of 0.25–16 µM for 72 h caused a significant reduction in the levels of pAKT and downstream p4EBP1, and p70S6K in BON1 and QGP-1 cells, respectively (Figure 5). Furthermore, WNT974 treatment also decreased pEGFR and pIGFR in BON1 and QGP-1 cells, and downregulated pERK in QGP-1 and NCI-H727 cells. pJNK decreased only in BON1 cells. These results further confirmed that the effects of WNT974 are cell-line-dependent (Figure 5).

### 3.5. Effects of the Selective β-Catenin Inhibitor PRI-724 on NET Cell Viability and Protein Expression

As the PORCN inhibitor WNT974 demonstrated inhibition of Wnt-signaling and antitumor activity in NET cells in vitro, we explored the effects of the β-catenin inhibitor PRI-724 as well as β-catenin siRNA on NET cells.

**Figure 6:**
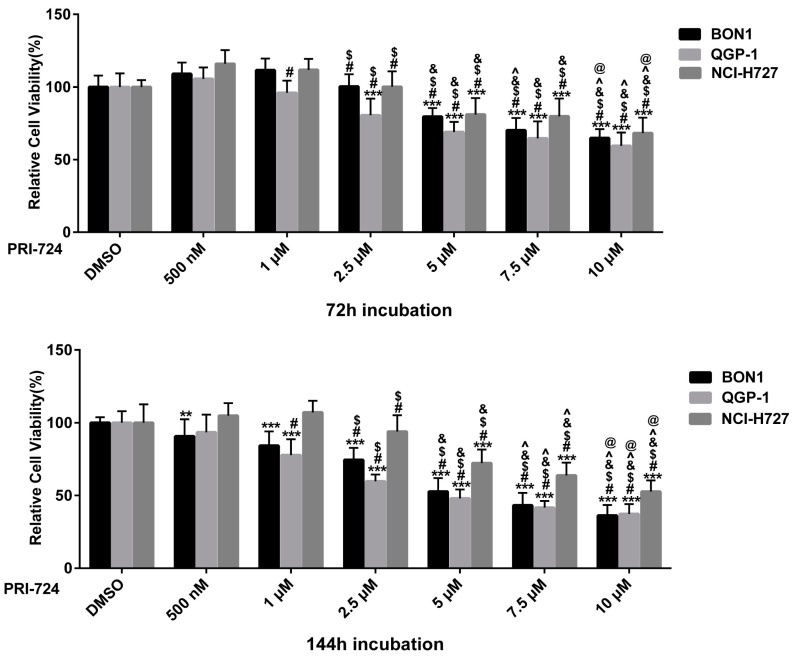
Effect of PRI-724 on the reduction of NET cell viability in a dose- and time-dependent manner. The cell viability of human pancreatic BON1 and QGP-1and bronchial NCI-H727 NET cell lines was assessed after treatment with PRI-724 compared with that of DMSO control. The data are expressed as mean ± SD. Each experiment, with technical triplicates, was repeated at least thrice. * *p* < 0.05, ** *p* < 0.01, and *** *p* < 0.001 compared with that of DMSO controls. ^#^ *p* < 0.05(1 μM–10 μM vs 500 nM respectively), ^$^ *p* < 0.05(2.5 μM–10 μM vs 1 μM respectively), ^&^ *p* < 0.05 (5 μM–10 μM vs. 2.5 μM respectively), ^ *p* < 0.05 (7.5 μM–10 μM vs 5 μM, respectively), ^@^ *p* < 0.05 (7.5 μM vs. 10 μM).

**Figure 7:**
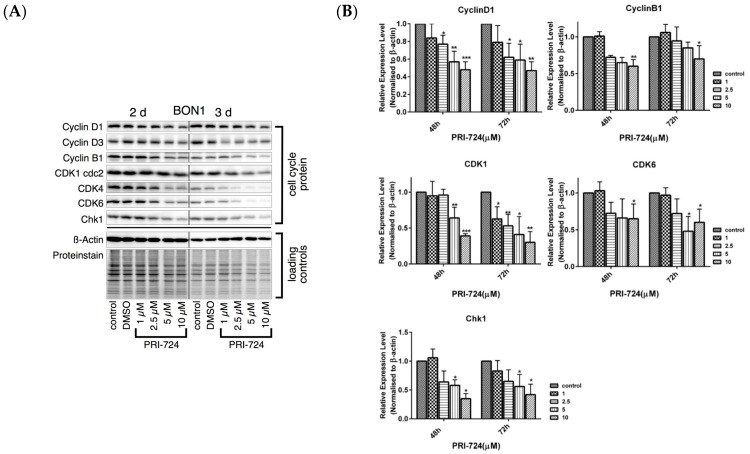
Effect of PRI-724 on protein expression levels of cell cycle proteins. BON1 cells were treated with PRI-724 for 48 h and 72 h and then subjected to Western blot analysis. (**A**) A representative Western blot is shown. Equal protein loading was verified in all Western blots by normalization to the total protein staining and by the housekeeping protein β-actin. (**B**) Densitometric quantification of Western blot data was performed. The DMSO control was set as 1.0. Relative expression levels (normalized to DMSO control) of treated cells were calculated in %. * *p* < 0.05, ** *p* < 0.01, and *** *p* < 0.001 compared with that of DMSO controls.

**Figure 8:**
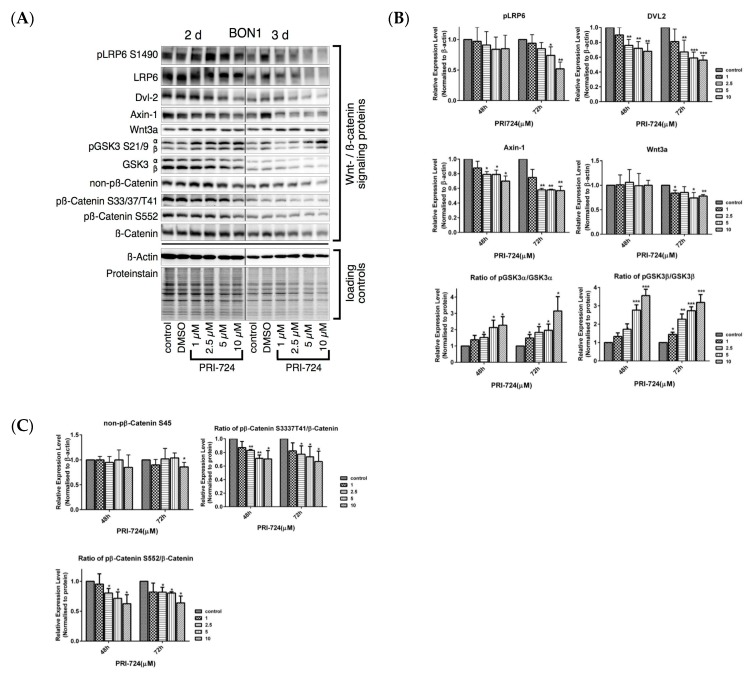
Effect of PRI-724 on protein expression levels of Wnt/β-catenin signaling proteins. BON1 cells were treated with PRI-724 for 48 h and 72 h and then subjected to Western blot analysis. (**A**) A representative Western blot is shown. Equal protein loading was verified in all Western blots by normalization to the total protein staining and by the housekeeping protein β-actin. (**B**,**C**) Densitometric quantification of Western blot data was performed. The DMSO control was set as 1.0. Relative expression levels (normalized to DMSO control) of treated cells were calculated in %. * *p* < 0.05, ** *p* < 0.01, and *** *p* < 0.001 compared with that of DMSO controls.

PRI-724, a selective inhibitor of the CBP/β-catenin interaction [11,12,13,39,40,41,42], caused a dose-dependent (concentrations from 0.5 to 10 µM) reduction in the viability of BON1, QGP-1, and NCI-H727 cells, respectively (Figure 6). PRI-724 caused a decrease in protein expression of cell proliferation and cell cycle-related proteins such as cyclinD1, CDK1, cyclinD3, CDK4, CDK6, CHK1 (Figure 7). As expected, PRI-724 treatment significantly downregulated the expression of β-catenin phosphorylation at S552 and at Ser33/37/T41, whereas there was no change in total β-catenin levels (Figure 8). PRI-724 also caused an increase in the expression of pGSK3 (Figure 8). These data demonstrate similar effects of PORCN inhibition by WNT974 (Figure 3 and Figure 4) and β-catenin inhibition by PRI-724 (Figure 7 and Figure 8) at the molecular level.

### 3.6. Effects of β-Catenin siRNA on the Regulation of NET Cell Viability and Protein Expression

**Figure 9:**
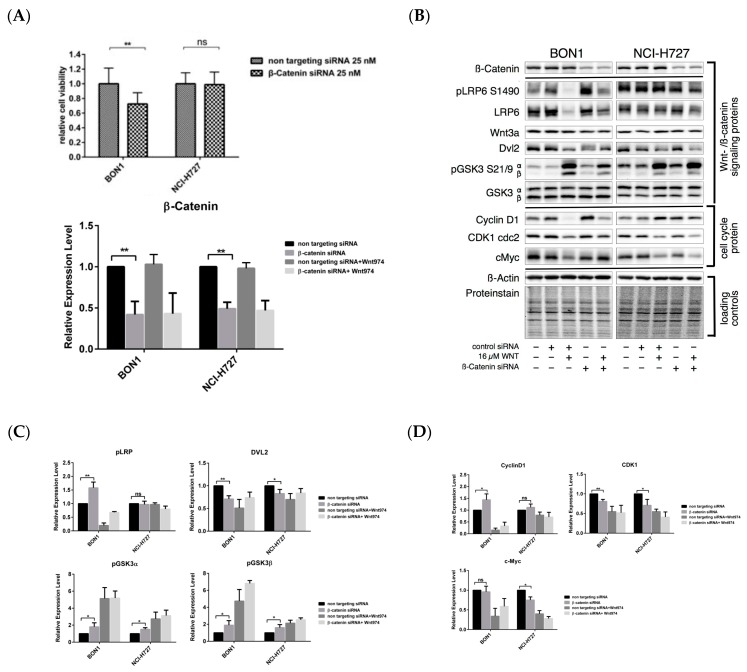
Effect of β-catenin siRNA on the reduction of NET cell viability and protein expression. BON1 and NCI-H727 cells transfected with β-catenin siRNA or non-targeting control (β-actin) siRNA for 72  h and then subjected to cell viability assay and Western blot analysis. (**A**) Cell viability assay and siRNA transfection efficacy. BON1 and NCI-H727 cells were transfected with β-catenin siRNA or non-targeting control (β-actin) siRNA for 72 h and then subjected to the cell viability assay. * *p* < 0.05 and ** *p* < 0.01 compared versus non-targeting control (β-actin) siRNA. Transfection with β-catenin siRNA caused downregulation of β-catenin protein expression, as shown by densitometric quantification of Western blot data. (**B**) Western blot. BON1 and NCI-H727 cells transfected with β-catenin siRNA or non-targeting control (β-actin) siRNA for 72 h and then subjected to Western blot analysis. A representative Western blot is shown. Equal protein loading was verified in all Western blots by normalization to the total protein staining and by the housekeeping protein β-actin. (**C**,**D**) Quantification of Western blot data of β-catenin siRNA with or without WNT974 for the regulation of NET cells. Densitometric quantification of Western blot data was performed. The DMSO control was set as 1.0. Relative expression levels (normalized to DMSO control) of treated cells were calculated in %. * *p* < 0.05 and ** *p* < 0.01 compared with that of non-targeting control (β-actin) siRNA control.

We transfected β-catenin siRNA to knock down β-catenin expression. The transfection of β-catenin siRNA decreased the protein expression of β-catenin in BON1 and NCI-H727 cells to 42 ± 16% and 49 ± 8%, compared to control transfection of non-targeting control (β-actin) siRNA (Figure 9A,B). β-Catenin siRNA significantly reduced the viability of BON1 cells (Figure 9A) but had no effect of the viability of NCI-H727 cells (Figure 9A). At the molecular level, the effects of the β-catenin siRNA were different from the effects of the PORCN inhibitor WNT974, e.g., regarding pLRP6, pGSK3, and cyclin D1 (Figure 9B,C).

We studied the effects of GSK3β siRNA on the regulation of NET cell viability and protein expression. In the canonical Wnt signaling cascade, GSK3 phosphorylates β-catenin at S33/S37/T41 and subsequently causes proteasomal degradation of β-catenin. Inhibition of GSK3 activity can activate canonical Wnt/β-catenin signaling [12,46,47].

**Figure 10:**
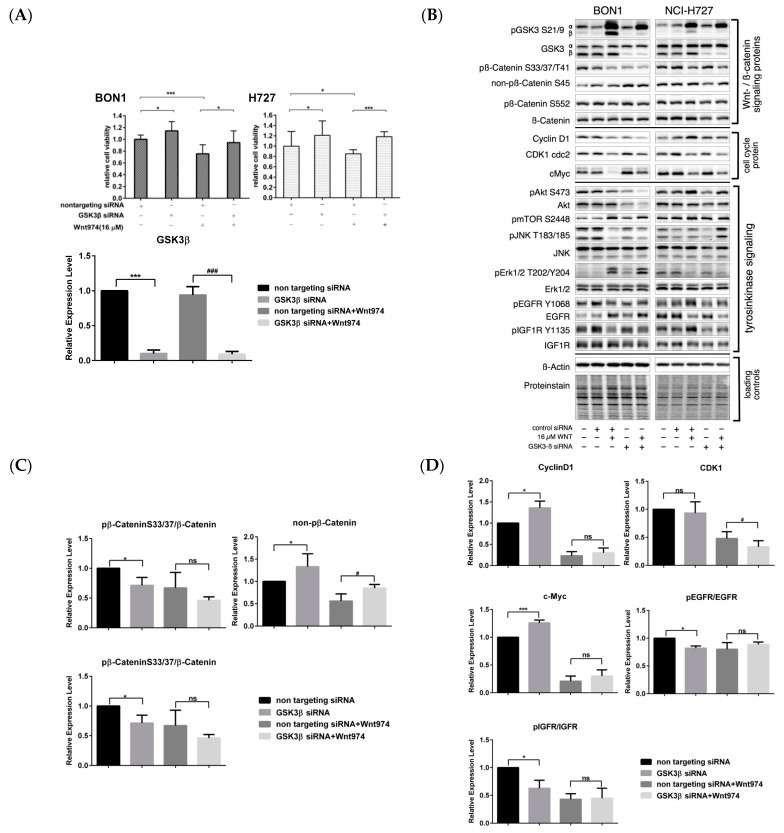
Effects of GSK3β siRNA on the regulation of NET cell viability and protein expression. BON1 and NCI-H727 cells were transfected with GSK3β siRNA or non-targeting control (β-actin) siRNA in the presence or absence of WNT974 for 72 h and then subjected to the cell viability assay and Western blot analysis. (**A**) Cell viability assay and siRNA transfection efficacy. BON1 and NCI-H727 cells were transfected with GSK3β siRNA or non-targeting control (β-actin) siRNA in the presence or absence of WNT974 for 72 h and then subjected to the cell viability assay. * *p* < 0.05 and *** *p* < 0.001 compared versus non-targeting control (β-actin) siRNA. Transfection with GSK3β siRNA caused downregulation of GSK3β protein expression, as shown by densitometric quantification of Western blot data. (**B**) Western blot. BON1 and NCI-H727 cells transfected with GSK3β siRNA or non-targeting control (β-actin) siRNA in presence or absence of WNT974 for 72 h and then subjected to Western blot analysis. A representative Western blot is shown. Equal protein loading was verified in all Western blots by normalization to the total protein staining and by the housekeeping protein β-actin. (**C**,**D**) Quantification of Western blot data of GSK3β siRNA with or without WNT974 for the regulation of BON1 cells. Densitometric quantification of Western blot data was performed. The DMSO control was set as 1.0. Relative expression levels (normalized to DMSO control) of treated cells were calculated in %. * *p* < 0.05 and *** *p* < 0.001 compared with that of non-targeting control (β-actin) siRNA control. ^#^ *p* < 0.05 and ^###^ *p* < 0.001 compared with WNT974 alone.

We transfected GSK3β siRNA to knock down GSK3β expression. The transfection of GSK3β siRNA significantly decreased the protein expression of GSK3β in BON1 and NCI-H727 cells to 10% ± 5% and 19% ± 4%, compared to control transfection of non-targeting control (β-actin) siRNA (Figure 10A,B). As expected, GSK3β knockdown significantly reduced β-catenin phosphorylation at Ser33/37/Thr41 in NET cells (Figure 10B) and also caused the upregulation of non-phosphorylated β-catenin S45 and of the downstream marker c-Myc (Figure 10B). GSK3β siRNA transfection caused a significant increase in NET cell viability (Figure 10A) and partially rescued BON1 and NCI-H727 cells from the WNT974-induced decrease in cell viability (Figure 10A).

On the other hand, phosphorylation of GSK3 is well known for inhibiting GSK3 enzyme activity [9,48,49,50,51]. WNT974 (Figure 4) caused strong upregulation of pGSK. The different crossways were further studied, and GSK3β siRNA with or without WNT974 downregulated pEGFR, pAKT, pERK, and pJNK, but upregulated mTOR slightly in the two cell lines, which confirmed that they are GSK3β-independent. pIGFR was also downregulated by GSK3β siRNA but was shown to be GSK3β-independent in NCI-H727 and GSK3β-dependent in BON1 (Figure 10B).

### 3.7. WNT974 Regulation of p21 and p53 Expression

Treatment with WNT974 at concentrations of 0.25–16 µM significantly decreased protein expression of p53 and p21 in BON1 cells, and of p21 in QGP1 cells (Figure S1).

### 3.8. WNT974 Regulation of Neurotensin and Menin Expression

Neurotensin (NT) is able to induce NET cell proliferation [17,19,52,53], and NT expression has been reported to be Wnt/β-catenin-dependent [17]. Menin is a tumor suppressor protein, and germline or somatic mutations of the multiple endocrine neoplasia type 1 (MEN1) gene are frequent in patients with pNETs [6,16,54]. Menin has been reported to regulate β-catenin [18].

Therefore, we investigated whether WNT974 can regulate the expression of either neurotensin (NT) or Menin. Western blot data showed expression of both NT and Menin to be modestly downregulated by WNT974 (Figure S2).

### 3.9. WNT974 Suppression of NET Cell Migration and Expression of the EMT Markers

To further understand WNT974 antitumor activity, we next determined the effect of WNT974 (8 and 16 µM) on regulation of the NET cell migration capacity, and found that WNT974 only modestly reduced the migration of BON1 and QGP-1 cells and had no effect on migration in NCI-H727 cells (Figure S3).

At the molecular level, we analyzed the expression of the mesenchymal marker vimentin and the epithelial marker E-cadherin, as well as the tight junction protein ZO-1, and found that WNT974 treatment decreased the expression level of vimentin and of ZO-1, whereas E-cadherin expression underwent no significant change in these cell lines (Figure S4).

## 4. Discussion

The current study demonstrates that the PORCN inhibitor WNT974 reduced NET cell viability in a dose- and time-dependent manner (Figure 1) and induced NET cell arrest at the G0/G1 and G2 phase of the cell cycle (Figure 2). At the molecular level, WNT974 treatment inhibited Wnt/β-catenin (Figure 3 and Figure 4) signaling, but also pAKT/mTOR, pEGFR and pIGFR (Figure 5) signaling in NET cells. In addition, treatment with the β-catenin inhibitor PRI-724 (Figure 6) inhibited NET cell viability in vitro. Treatment with β-catenin siRNA (Figure 9) showed cell-line-specific effects. These data indicate canonical Wnt/β-catenin signaling (Figure 3 and Figure 4) and non-canonical Wnt/receptor tyrosine kinase signaling (Figure 5) to be involved in NET tumor cell growth regulation. These data suggest that targeting of Wnt/β-catenin signaling might be a novel molecular targeted therapeutic strategy against NETs.

The potential role of Wnt/β-catenin signaling in neuroendocrine tumors (NETs) has recently been reviewed [9,16]. Menin seems to be a negative regulator of β-catenin, and in MEN1-deficient knockout mice, β-catenin is activated in pNETs while, vice versa, conditional β-catenin knockout decreased tumorigenesis of pNETs in this in vivo model [18].

Analysis of human NET tumor samples demonstrated mutations of the MEN1 gene in up to 35% of lung carcinoids [21,22,23] and up to 37% of pNETs [6]. Mutations in other well-known negative regulators of the Wnt/β-catenin signaling cascade, such as the APC gene, were present in 6–12% of typical/atypical lung carcinoids [24] and in 8%–23.0% of SI-NETs [25,26]. A single nucleotide variation in the APC gene has been reported in QGP-1 cells but has not been found in BON1 and NCI-H727 cells [55]. Further experiments in NET cell lines with MEN1 silencing or in MEN1-deficient knockout mice [18] should be performed to evaluate the potential effect of inhibition of Wnt/β-catenin signaling in MEN1-deficient tumors due to somatic or germline MEN1 mutations. Therefore, there might be a subgroup of patients with neuroendocrine tumors who are responsive to personalized molecular targeted therapy with inhibitors of Wnt/β-catenin signaling. Further clinical studies are needed to define these subgroups for individualized precision oncology.

We revealed that the expression of pLRP6, DVL2, and Wnt5a/b was downregulated by WNT974 in NET cells (Figure 4), and correspondingly, we also found that WNT974 treatment downregulated the level of β-catenin phosphorylation at Ser33/Ser37/Thr41 (Figure 4). These findings indicate that, in NET cells, the Wnt/β-catenin canonical pathway is altered by WNT974. Axin is a concentration-limiting factor in the β-catenin degradation complex. Our current study showed that, with WNT974 treatment in NET cells, Axin1 expression initially showed a time-dependent increase, but subsequently decreased (Figure 4).

Cell cycle progression is tightly controlled by a complex of cell cycle regulatory molecules, such as cyclin-dependent kinases (CDKs), CDK inhibitors, and cyclins. Indeed, Wnt/β-catenin signaling controls cell proliferation and migration, and an increase in β-catenin expression or activity will initiate transcriptional activation of cyclinD1/D3, CDK1/2/4, and c-Myc proteins, which control the cell cycle transition from G1 to S phase [56,57]. In the current study, we were able to show that WNT974 induced cell cycle arrest at the G1 or G2 phase of NET cells (Figure 2A–C) but did not induce NET cells to undergo apoptosis as demonstrated by sub-G1 events (Figure 2D) and caspase 3/7 assay activity (Figure 2E). Previous reports of the induction of cell cycle regulation and apoptosis by WNT974 have been inconsistent. While Boone JD et al. [34] reported cell cycle arrest to only to be induced in primary ovarian cancer following WNT974 treatment, a study by Tian et al. [38] reported that WNT974 enhanced apoptosis in HepG2 cells. Our results in NET cells demonstrating only cell cycle inhibition, and no apoptosis, suggest that a combination of WNT974 with other molecular inhibitors or chemotherapy might be reasonable to effectively control NETs or other cancers [37,38].

Interestingly, incubation with WNT974 caused a decrease in the expression of p53 and p21 proteins (Figure S1). At first glance, this seems to be in contrast with WNT974 inducing cell cycle arrest in NET cells as we have previously demonstrated that upregulation of the tumor suppressor p53 and the cyclin-dependent kinase inhibitor p21 were important in NET cell cycle regulation and growth inhibition [58]. However, the interplay between p53–p21 and Wnt/β-catenin is not fully understood and might reveal differential effects on cancer cells [59,60], and p21 has also been suggested to have dual/differential effects in cancer cells, causing cell cycle arrest but also anti-apoptotic effects [61,62,63,64,65].

Inhibitory effects of WNT974 on the non-canonical Wnt/receptor tyrosine kinase signaling pathway (Figure 5) and on GSK3 activity (Figure 4, Figure 9B and Figure 10B) in NETs might contribute to its antiproliferative efficacy in NET cells in vitro.

WNT974 decreased the expression of pEGFR, pIGFR, pAKT, pERK, and pJNK in a GSK3β-independent and cell-line-dependent manner (Figure 5).

GSK3 has been implicated in the pathogenesis of various diseases, including cancer [9]. GSK3 can act paradoxically as a tumor suppressor gene in some cancer types but as an oncogene in others [9]. Phosphorylated GSK3 is the inactive form of GSK3 [9]. WNT974 (Figure 4, Figure 9B and Figure 10B) caused a strong upregulation of pGSK3, thus indicating inactivation of GSK3. We have previously demonstrated that inhibition of GSK3 in neuroendocrine tumor cells results in potent antiproliferative effects [48,49,50,51].

On the other hand, GSK3 is known to play a pivotal role in regulating the canonical Wnt pathway [12]. In the canonical Wnt signaling cascade, GSK3 phosphorylates β-catenin at S33/S37/T41 and subsequently causes proteasomal degradation of β-catenin. Inhibition of GSK3 activity can activate canonical Wnt/β-catenin signaling [12,46,47]. Accordingly, GSK3β siRNA significantly enhanced the viability of BON1 and NCI-H727 cells (Figure 10), while GSK3β knockdown downregulated the level of phosphorylated β-catenin, but upregulated the expression of non-phosphorylated β-catenin (Figure 10). Our results with GSK3β siRNA might be limited as only GSK3β but not GSK3α was knocked down in our experiments, while others have demonstrated that the downregulation of GSK3α and GSK3β was necessary to obtain a functional knockdown [46]. Our findings indicate that the WNT974-mediated inhibition of NET cell viability might occur through direct inhibition of GSK3β signaling. However, as complex bidirectional regulatory mechanisms exist between GSK3 and Wnt/β-catenin signaling [66,67], a counter-regulatory mechanism of the NET cells due to WNT974-mediated inhibition of canonical Wnt/β-catenin signaling might also cause the inhibition of GSK3 activity as a rescue mechanism to reestablish β-catenin signaling.

The β-catenin inhibitor PRI-724 inhibited NET cell viability in vitro (Figure 6) and caused downregulation of cell cycle proteins as cyclinD1, CDK1, and CHK1 (Figure 7A,B). The PORCN inhibitor WNT974 (Figure 1, Figure 2, Figure 3 and Figure 4) and further downstream β-catenin inhibitor PRI-724 (Figure 6 and Figure 7) demonstrated similar effects on NET cell proliferation (Figure 1; Figure 6) and on pGSK3 (Figure 4 and Figure 8) and cell cycle proteins (Figure 3 and Figure 7). Also treatment with β-catenin siRNA (Figure 9) in BON1 cells decreased NET cell viability (Figure 9A). However, in contrast to the significant effects on pLRP6, cyclin D1, and CDK1 expression by WNT974 (Figure 3 and Figure 4) and by PRI-724 (Figure 7 and Figure 8), β-catenin siRNA (Figure 9B,C) showed no significant effects. Thus, the molecular mechanisms of action of the β-catenin inhibitor PRI-724 and β-catenin siRNA seem somehow different, however, this finding might be limited by the fact that β-catenin siRNA caused only a partial decrease of β-catenin protein expression (Figure 9A).

Our in vitro study has several limitations. A major limitation is the limited number of human neuroendocrine cell lines investigated, and the fact that the established human neuroendocrine tumor cell lines differ from neuroendocrine tumors in vivo with respect to tumor genetics, tumor biology, proliferation rate, and population doubling time [9,45,55,68]. The WNT974 concentrations that were effective in the investigated NET cell lines were rather high compared to other more WNT974-sensitive cancer cell lines [32]. Future studies on WNT/β-catenin signaling in neuroendocrine tumors should also aim to investigate the effects in primary neuroendocrine tumor cell cultures, as has been established [45,69], but this was beyond the current scope of this work.

## 5. Conclusions

In conclusion, the PORCN inhibitor WNT974 and the β-catenin inhibitor PRI-724 exert antiproliferative activities in human NET tumor cell lines in vitro. WNT974 inhibits canonical Wnt/β-catenin signaling and exerts an inhibitory effect on the non-canonical Wnt/receptor tyrosine kinase signaling pathways, PI3K/AKT/mTOR, EGFR, and IGFR, as well as on GSK3 activity. All these mechanisms might be involved in the inhibition of NET tumor cell growth and, in future, the molecular mechanisms of WNT974 on NET tumor cells need further in-depth investigation. Targeting Wnt/β-catenin signaling by various approaches, such as through the use of PORCN inhibitors or direct β-catenin inhibitors, might be a promising molecular targeted therapeutic strategy in NETs. Potential subgroups of patients with neuroendocrine tumors who may actually be responsive to personalized molecular targeted therapy based on inhibitors of Wnt/β-catenin signaling [16] still need to be defined. Further preclinical studies and clinical trials are also needed.

## Supplementary Materials

The following are available online at https://www.mdpi.com/2072-6694/12/2/345/s1, Figure S1: Effect of WNT974 on protein expression of p53 and of p21, Figure S2: Effect of WNT974 on protein expression of neurotensin and of Menin, Figure S3: Effect of WNT974 on regulation of NET cell migration, Figure S4: Effect of WNT974 on protein expression of epithelial to mesenchymal transition (EMT) markers.
