# Supplementary material for: Inhibition of Wnt/β-Catenin Signaling in Neuroendocrine Tumors In Vitro: Antitumoral Effects
Source: Cancers (Basel). 2020 Feb 4;12(2):345. doi: 10.3390/cancers12020345 (PMC7072467; doi:10.3390/cancers12020345)
Supplement: Supplementary file 1 [file cancers-12-00345-s001.pdf]

cancers

Article

Inhibition of Wnt/β-Catenin Signaling in Neuroendocrine Tumors in vitro: Antitumoral Effects

Xi-Feng Jin, Gerald Spöttl, Julian Maurer, Svenja Nölting and Christoph Josef Auernhammer

Supplementary Materials

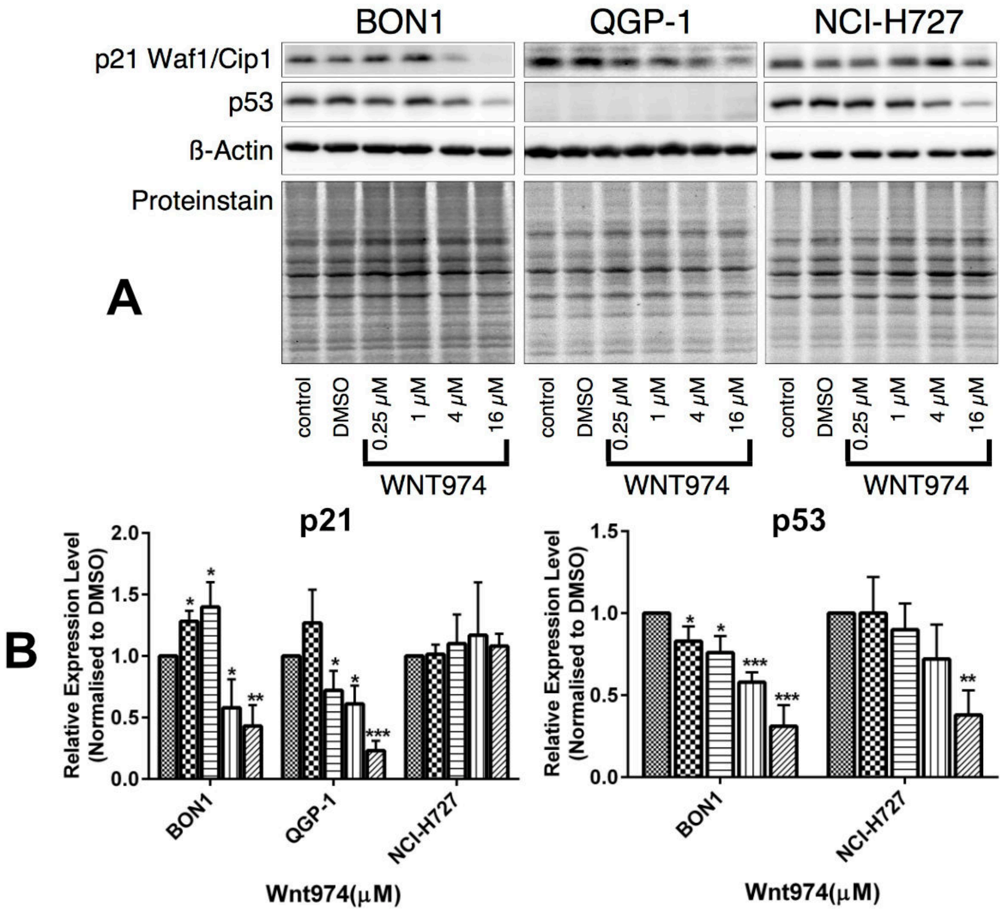

Supplementary Figure 1. Effect of WNT974 on protein expression of p53 and of p21. BON1, QGP-1, and NCI-H727 cells were treated with or without WNT974 for 72 h and then subjected to Western blot analysis. (**A**) A representative Western blot is shown. Equal protein loading was verified in all Western blots by normalization to the total protein staining and by the housekeeping protein β-actin**.** (**B**) Densitometric quantification of Western blot data was performed. The DMSO control was set as 1.0. Relative expression levels (normalized to DMSO control) of treated cells were caluclated in %. * $p < 0.05$, ** $p < 0.01$, and *** $p < 0.001$ compared with that of DMSO controls.

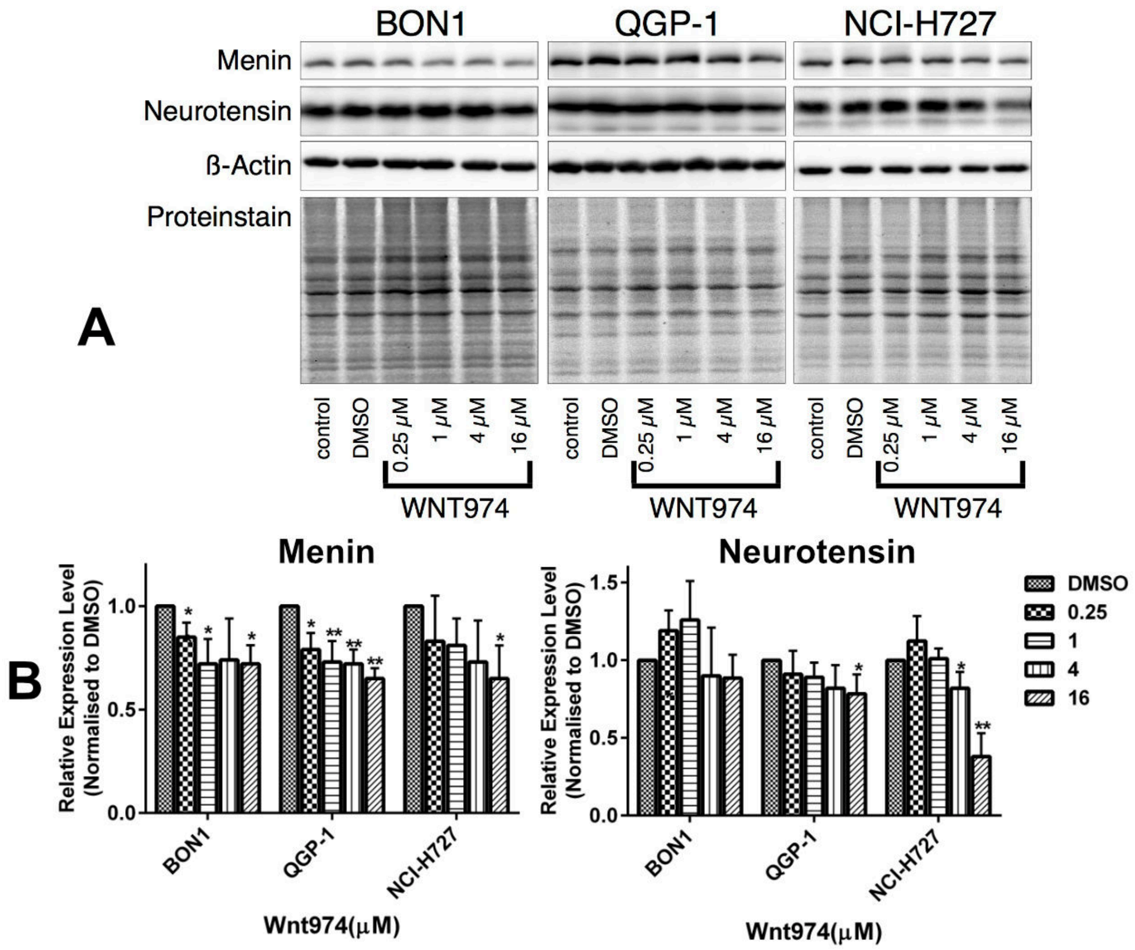

Supplementary Figure 2. Effect of WNT974 on protein expression of neurotensin and of Menin. BON1, QGP-1, and NCI-H727 cells were treated with or without WNT974 for 72 h and then subjected to Western blot analysis. (**A**) A representative Western blot is shown. Equal protein loading was verified in all Western blots by normalization to the total protein staining and by the housekeeping protein β-actin. (**B**) Densitometric quantification of Western blot data was performed. The DMSO control was set as 1.0. Relative expression levels (normalized to DMSO control) of treated cells were calculated in %. * $p < 0.05$, ** $p < 0.01$, and *** $p < 0.001$ compared with that of DMSO controls.

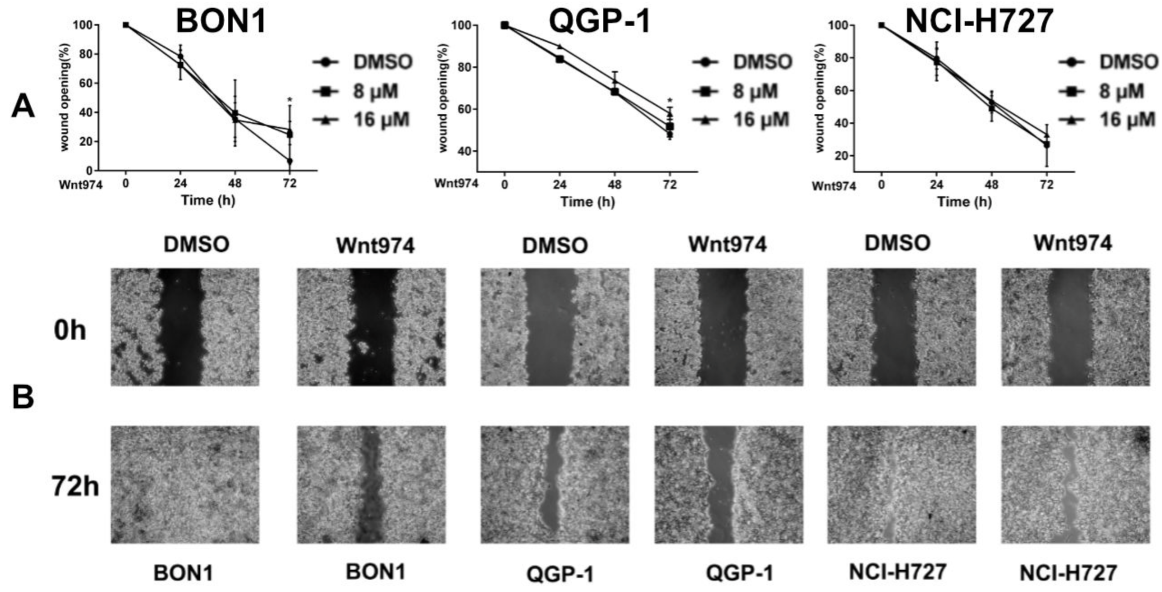

Supplementary Figure 3. Effect of WNT974 on regulation of NET cell migration. Wound healing assay. BON1, QGP-1, and NCI-H727 cells were treated with or without WNT974 for 72 h and then subjected to wound healing assay. (**A**) Quantitative analysis of the percentage of open wound area in the DMSO control group in comparison to WNT974 treated cells at 0, 24, 48 and 72 h. The mean values were normalized against the woundwidth at time 0. (**B**) Representative photomicrographs of NET cell migration at the highest investigated dose of WNT974 (16 μM).

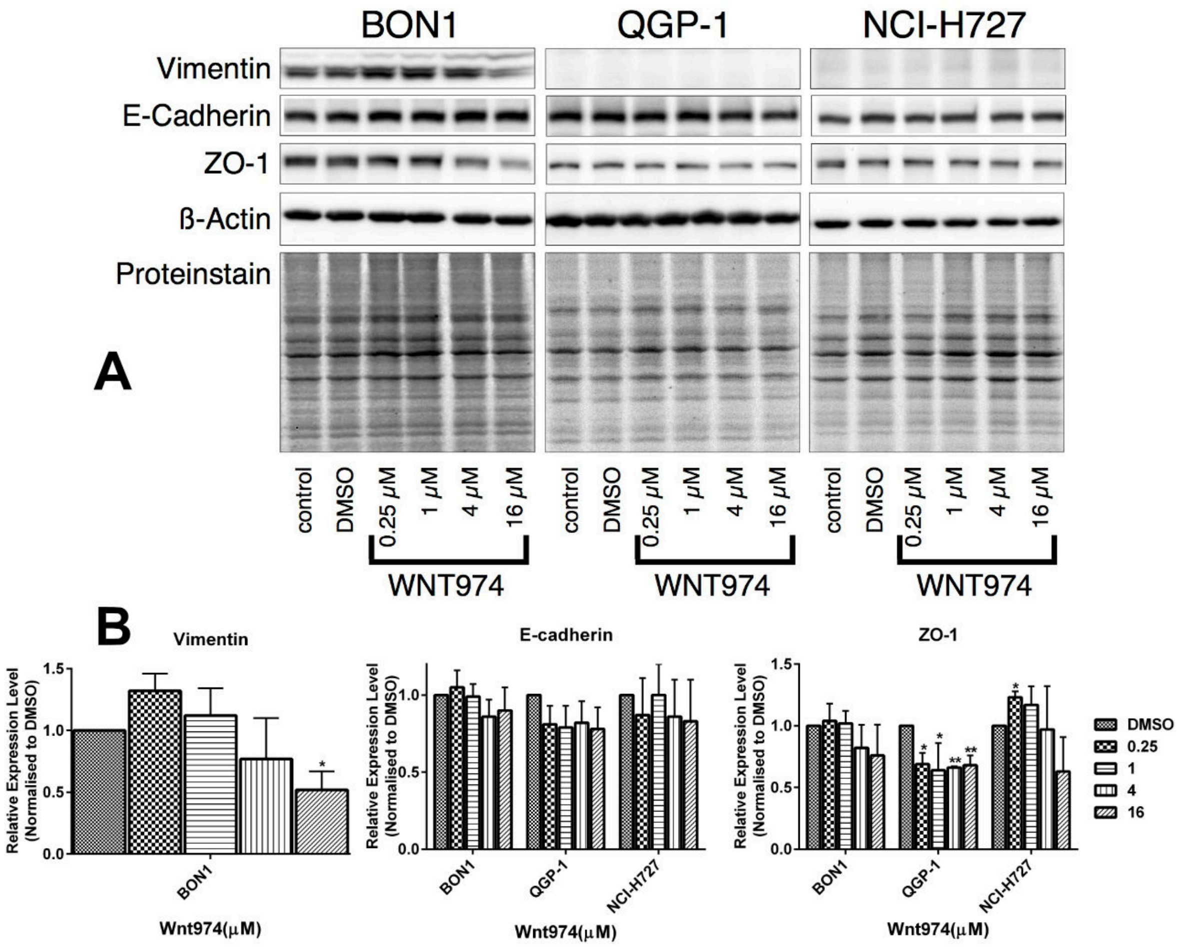

Supplementary Figure 4. Effect of WNT974 on protein expression of epithelial to mesenchymal transition (EMT) markers. BON1, QGP-1, and NCI-H727 cells were treated with or without WNT974 for 72 h and then subjected to Western blot analysis. (**A**) A representative Western blot is shown. Equal protein loading was verified in all Western blots by normalization to the total protein staining and by the housekeeping protein β-actin**.** (**B**) Densitometric quantification of Western blot data was performed. The DMSO control was set as 1.0. Relative expression levels (normalized to DMSO control) of treated cells were calculated in %. * $p < 0.05$, ** $p < 0.01$, and *** $p < 0.001$ compared with that of DMSO controls.

© 2020 by the authors. Licensee MDPI, Basel, Switzerland. This article is an open access article distributed under the terms and conditions of the Creative Commons Attribution (CC BY) license (http://creativecommons.org/licenses/by/4.0/).
